# Persistent homology for MCI classification: a comparative analysis between graph and Vietoris-Rips filtrations

**DOI:** 10.3389/fnins.2025.1518984

**Published:** 2025-02-26

**Authors:** Debanjali Bhattacharya, Rajneet Kaur, Ninad Aithal, Neelam Sinha, Thomas Gregor Issac

**Affiliations:** ^1^Department of Artificial Intelligence, Amrita School of Artificial Intelligence, Amrita Vishwa Vidyapeetham, Bengaluru, India; ^2^Centre for Brain Research, Indian Institute of Science (IISc), Bengaluru, Karnataka, India; ^3^Vision and Artificial Intelligence Lab, Indian Institute of Science (IISc), Bengaluru, Karnataka, India

**Keywords:** mild cognitive impairment, fMRI time-series, persistent homology, graph filtration, Vietoris-Rips filtration, Wasserstein distance, classification

## Abstract

**Introduction:**

Mild cognitive impairment (MCI), often linked to early neurodegeneration, is associated with subtle disruptions in brain connectivity. In this paper, the applicability of persistent homology, a cutting-edge topological data analysis technique is explored for classifying MCI subtypes.

**Method:**

The study examines brain network topology derived from fMRI time series data. In this regard, we investigate two methods for computing persistent homology: (1) Vietoris-Rips filtration, which leverages point clouds generated from fMRI time series to capture dynamic and global changes in brain connectivity, and (2) graph filtration, which examines connectivity matrices based on static pairwise correlations. The obtained persistent topological features are quantified using Wasserstein distance, which enables a detailed comparison of brain network structures.

**Result:**

Our findings show that Vietoris-Rips filtration significantly outperforms graph filtration in brain network analysis. Specifically, it achieves a maximum accuracy of 85.7% in the Default Mode Network, for classifying MCI using in-house dataset.

**Discussion:**

This study highlights the superior ability of Vietoris-Rips filtration to capture intricate brain network patterns, offering a robust tool for early diagnosis and precise classification of MCI subtypes.

## 1 Introduction

Mild cognitive impairment (MCI) has become a prominent subject of investigation and clinical attention as it represents an intermediate stage between the anticipated cognitive decline associated with normal aging and the more severe cognitive and functional deficits observed in dementia (Mosti et al., 2019; Petersen and Negash, 2008). MCI is characterized by a measurable decline in cognitive abilities, such as memory, language, or executive function. This decline is greater than expected for an individual's age and education level. However, it does not significantly interfere with their ability to perform everyday activities (Gauthier et al., 2006; Knopman and Petersen, 2014). It is prevalent in older adults. While some individuals with MCI remain stable or even return to normal cognitive function over time, over half of them progress to dementia within 5 years (Gauthier et al., 2006). Therefore, MCI can be viewed as a potential precursor to dementia.

In recent years, there have been notable advancements in brain imaging, particularly with functional MRI (fMRI), which provide valuable insights into the impact of MCI on brain function. By analyzing changes in different brain networks, we can enhance our understanding of how MCI disrupts communication within the brain. These disruptions are crucial in detecting the disease at an early stage, potentially allowing for interventions that can slow down or reverse cognitive decline associated with MCI and its sub-types. However, the fMRI data analysis often faces challenges such as high dimensionality, inter-subject variability, and the difficulty in capturing non-linear and dynamic changes in brain connectivity. Numerous studies have attempted to distinguish MCI and its sub-types, often with moderate success in terms of classification accuracy (Kam et al., 2018; Jie et al., 2018b,a; Kam et al., 2020; Wang et al., 2020; Lee et al., 2021; Yang et al., 2021). Conventional methods largely rely on static connectivity metrics and predefined brain network structures, which may overlook subtle topological changes and higher-order interactions in brain networks. Unlike traditional methods, our approach introduces an innovative computational topology-based technique. While most state-of-the-art methods, including deep learning and network-based approaches, primarily analyze spatial and temporal features or depend on predefined connectivity metrics, our method uncovers deeper, intrinsic properties of brain networks, offering a more comprehensive understanding of their topological structure. Given the limitations of conventional methods in capturing the complex topological changes in brain networks associated with neurodegenerative diseases, there is a need for more robust approaches. This study aims to leverage persistent homology, a technique from computational topology, to extract stable and highly discriminative topological features from fMRI-derived brain networks. By identifying higher-order interactions and subtle network alterations, this approach provides a novel perspective on brain connectivity and enhances the classification accuracy of MCI subtypes, ultimately contributing to the development of more effective biomarkers for early diagnosis and disease monitoring.

Persistent homology is a powerful approach within the field of algebraic topology that falls under the umbrella of topological data analysis. It offers a robust framework for examining the topological properties of data, particularly in relation to shape and structure. The objective of persistent homology is to trace how topological features on a given space appear and disappear as the scale value gradually changes. By capturing the underlying structure and relationships within complex medical data, persistent homology holds great promise for generating new insights and improving the accuracy of clinical decision-making. Notably, its application has been observed in the analysis of Autism Spectrum Disorder (Jafadideh and Asl, 2022), Schizophrenia (Stolz et al., 2021), and brain tumor analysis (Bhattacharya et al., 2025). Moreover, it has been utilized to examine differences in visual brain networks (Bhattacharya et al., 2023). The current study is an extension of our previous work (Aithal et al., 2025), where we leveraged persistent homology using Vietoris-Rips filtration for the differential diagnosis of MCI. In this work, we aim to rigorously compare the effectiveness of graph filtration with the previously utilized Vietoris-Rips filtration. In the previous study Wasserstein distance matrices computed from persistent homology at different dimensions are used as feature for classification. Contrary to this, in the present study we hypothesize that persistent homology, particularly when utilizing raw persistence features generated from graph filtration can effectively differentiate between healthy individuals and those at various stages of MCI, including Early and Late MCI. However, our findings reveal that while graph filtration offers valuable insights into the topological structure of brain connectivity, the classification results using Vietoris-Rips filtration are consistently superior. The Vietoris-Rips approach not only surpasses graph filtration in distinguishing MCI subtypes but also outperforms many state-of-the-art methods in MCI classification. These results highlight the greater efficacy of Vietoris-Rips filtration in extracting meaningful topological features, making it a more robust tool for clinical applications in early MCI detection and diagnosis. This findings of this study provide crucial insights into the comparative strengths of these two filtration approaches. Vietoris-Rips filtration proves to be more robust in capturing the intricate topological patterns in brain connectivity that are essential for accurate diagnosis. This superiority reinforces its potential for use in clinical settings, offering a more reliable framework for early detection and classification of MCI subtypes. Additionally, this research highlights the importance of choosing the appropriate filtration method in persistent homology applications, contributing new knowledge to the field of topological data analysis for disease classification. Our work serves as a guide for future studies, emphasizing that the choice of filtration has a significant impact on the outcomes of persistent homology-based classification.

## 2 Materials and methods

### 2.1 Dataset description

The study employs fMRI images from two distinct population cohorts. The baseline analysis utilizes subjects from the publicly available Alzheimer's Disease Neuroimaging Initiative (ADNI) dataset (Jack et al., 2008), characterized by a repetition time (TR) of 3000 ms and an echo time (TE) of 30 ms. This is complemented by our in-house cohort from the TATA Longitudinal Study for Aging (TLSA), which features a TR of 3200 ms and a TE of 30 ms. The TLSA cohort, an urban study, is dedicated to the long-term investigation of risk and protective factors associated with dementia in India. Both cohorts used sagittal plane imaging with 3D acquisition. Participants diagnosed with MCI had no underlying neurodegenerative diseases besides MCI itself, while healthy subjects had no previous instances of cognitive impairment, stroke, or significant psychiatric disorders. The ADNI cohort includes the following groups: EMCI (*N* = 162, M:F = 59:103, Age: 72.3 ± 6.7), LMCI (*N* = 141, M:F = 86:55, Age: 72.1 ± 7.8), and healthy control (HC: *N* = 177, M:F = 81:96, Age: 75.1 ± 6.3). The efficacy of our proposed methodology is further evaluated using our in-house TLSA cohort, which consists of gender and age-matched MCI (*N* = 35, M:F = 20:15, Age: 63.9 ± 9.04) and HC (*N* = 35, M:F = 20:15, Age: 63.8 ± 9.1) subjects. In our in-house MCI cohort, individuals were diagnosed with MCI according to specific criteria, including a Clinical Dementia Rating (CDR) score of 0.5, which serves as the current gold standard for assessing the stages of dementia.^1^ The demographic details and inclusion criteria of the two cohorts are tabulated in Table 1. The study utilizes fMRI time series from Dosenbach Regions of Interest (ROIs), encompassing a total of 160 ROIs selected from six classical brain networks. All fMRI images were processed using a consistent preprocessing pipeline, which included motion correction, slice timing adjustment, normalization to the standard MNI space, and regression to account for nuisance variables. These preprocessing steps were conducted using FMRIB Software Library (FSL) version 6.0.6 (Jenkinson et al., 2012).

**Table 1:** Demographic details and inclusion criteria of the two cohorts.

**Demographic**	**ADNI data**			**TLSA data**	
**Details**	**HC**	**EMCI**	**LMCI**	**HC**	**MCI**
No. of subjects	177	162	141	35	35
Gender (M:F)	81:96	59:103	86:55	20:15	20:15
Age	75.1 ± 6.3	72.3 ± 6.7	72.1 ± 7.8	63.7 ± 9.1	63.8 ± 9.0
CDR global	0	0.5	0.5	0	0.5

### 2.2 Overview on proposed methodology

**Figure 1:**
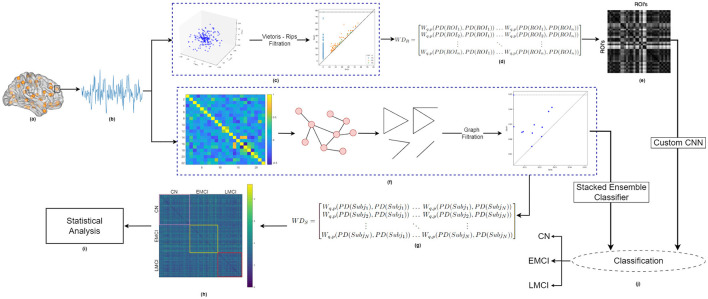
Block diagram showing the Proposed Methodology. **(A)** Brain schematic with Dosenbach ROIs, **(B)** generated fMRI timeseries for one specific ROI of a specific brain network, **(C)** Persistence diagram as obtained for one representative subject using Vips-rips filtration applied on 3D Point cloud, for a specific brain network, **(D)** Subject-specific inter-ROI Wasserstein distance matrix, **(E)** Visualization of inter-ROI Wasserstein distance, **(F)** Persistence diagram obtained for the same subject using graph filtration applied to the partial correlation matrix of the fMRI time series for the specific brain network, **(G)** ROI-specific inter-subject Wasserstein distance matrix, **(H)** Visualization of inter-subject Wasserstein distance, **(I)** Statistical analysis of inter-subject Wasserstein distance, **(J)** Classification framework: (i) stack ensemble classifier used for classification using graph-filtration-based top 10 most persistent features for each homology dimension, and (ii) custom CNN used for classification using subject-specific inter-ROI Wasserstein distance, for each homology dimension.

The block diagram illustrating the proposed methodology is shown in Figure 1. This study compares the classification performance achieved using two different filtration choices for constructing persistence diagrams: (i) Vietoris-Rips filtration and (ii) graph filtration. The analysis begins by extracting time series data from resting-state fMRI volumes. To generate persistence diagrams using Vietoris-Rips filtration, the 1D fMRI time series is first transformed into a 3D point cloud. In contrast, for graph filtration, correlation analysis is performed directly on the extracted 1D fMRI time series data, using both marginal and partial correlations to construct a positively correlated graph from distinct brain regions (nodes). The adjacency matrix generated from this graph is then used to compute persistence diagrams via graph filtration. To quantify topological changes, we employ the Wasserstein distance metric. These changes are analyzed in two distinct ways: (i) across subjects for a specific region of interest (ROI) when using persistence diagrams generated from graph filtration, and (ii) across ROIs for a given subject when using persistence diagrams derived from Vietoris-Rips filtration. Finally, the classification of healthy individuals, EMCI, and LMCI is carried out using two feature sets: (a) the top ten most persistent homology features obtained from graph filtration, and (b) inter-ROI Wasserstein distance features derived from the Vietoris-Rips filtration. Each step of the proposed methodology is described in detail in the following sub-sections.

### 2.3 Extraction of fMRI time series

**Figure 2:**
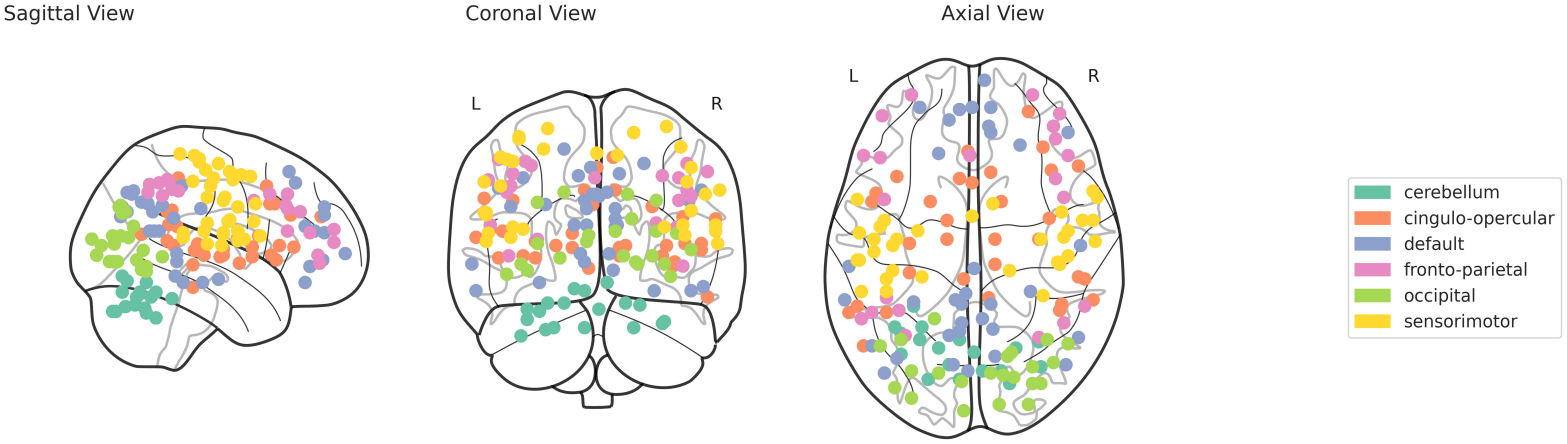
Visualization of Dosenbach's Regions of Interest (ROIs) for the six brain networks.

The fMRI time series provides insights into the temporal dynamics of brain activity, allowing for an in-depth analysis of how various brain regions interact over time. In this study, specific brain regions are carefully selected from Dosenbach's ROIs to extract fMRI time series, aiming to identify significant patterns and differences between MCI sub-types and healthy controls. The Dosenbach's ROIs (Dosenbach et al., 2010), consisting of 160 regions, are partitioned into six distinct brain networks: cerebellum (CB) comprising 18 nodes, cingulo-opercular (CO) with 32 nodes, default mode network (DMN) consisting of 34 nodes, fronto-parietal (FP) comprising 21 nodes, occipital (OP) with 22 nodes, and sensorimotor (SM) consisting of 33 nodes. Figure 2 displays the different ROIs for these six distinct networks. These networks encompass various interconnected brain regions, each associated with particular cognitive, sensory, and motor functions. Given that these functions may exhibit unique disruption patterns at different stages of MCI, the analysis of all six networks offers a comprehensive assessment of network-specific changes. These changes could potentially serve as distinct biomarkers for different stages or types of cognitive impairment. A representative 5 mm radius sphere centered at each voxel location was used to generate the time series *v*_*t*_, *t* = 1, 2, ..., *N*. The fMRI preprocessing was carried out using FSL's FEAT. Briefly, the steps involved discarding the initial 10 volumes, applying MCFLIRT for motion correction, performing brain extraction, applying a 5 mm spatial smoothing kernel, and conducting high-pass temporal filtering. Additionally, the fMRI data were registered (using a 12-degree-of-freedom linear transformation) to the corresponding structural image and then to MNI152 space. As per standard practices, the mean contributions of motion correction parameters, as well as CSF and WM signals—treated as nuisance variables—were regressed out during preprocessing.

### 2.4 Persistent homology using Vietoris-Rips filtration

Efficiently creating a point cloud representation from 1D time series data is a crucial step in computing persistent homology (Perea and Harer, 2015; Gakhar and Perea, 2024), which is essential for analyzing the intrinsic topological properties of MCI. To achieve this, the study uses sliding window embedding (SW) with an embedding dimension *M* = 2 and a time lag τ = 1, converting the fMRI time series *v*_*t*_ into 3D point clouds *S*.

#### 2.4.1 Sliding window embedding

In order to construct a sliding window embedding from a 1D time series, we start by taking the original time series, *f*(*t*) = {*f*_1_, *f*_2_, …, *f*_*N*_}, where each value corresponds to a data point at a specific time *t*. The sliding window embedding involves selecting a window of fixed size (determined by the embedding dimension *M*) and a time lag τ. For example, with an embedding dimension *M* = 2 and time lag τ = 1, each time step *t* is transformed into a 3D point by selecting the values at three consecutive time points: *f*(*t*), *f*(*t* + τ), and *f*(*t* + 2τ). This results in the point (*f*(*t*), *f*(*t* + τ), *f*(*t* + 2τ)). As the window slides across the time series from *t* = 1 to *t* = *N* − *M*, a sequence of such 3D points is generated, forming a point cloud that represents the time series in a higher-dimensional space. The sliding window process captures the temporal structure of the data and allows the construction of a point cloud that can be used for further analysis, such as computing persistent homology or studying the dynamic behavior of the underlying system. In this study, the sliding window length is set to 3, minimizing noise and enhancing interpretability. Mathematically, the sliding window embedding of a function *f* based at *t* ∈ ℝ into ℝ^*M* + 1^ is represented as follows (Equation 1):


(1) $$\begin{array}{l} {\mathrm{SW}}_{M,\tau }f:\mathbb{R}\to {\mathbb{R}}^{\left(M+1\right)},\;t\to \left[\begin{array}{c} f\left(t\right)\\ f\left(t+\tau \right)\\ f\left(t+2\tau \right) \end{array}\right] \end{array}$$

By selecting different values of *t*, a sliding window point cloud is generated, representing the function *f* in 3D space. This approach is supported by literature indicating the effectiveness of sliding window methods in capturing dynamic functional connectivity in rs-fMRI. In this study, for embedding dimension *M* = 2, the point cloud of the fMRI time series is represented by Equation 2:


(2) $$\begin{array}{l} S=\left\{v_{i}:i=1,\ldots ,N,\;v_{i}\in {\mathbb{R}}^{3}\right\} \end{array}$$

Each point *v*_*i*_ in the point cloud *S* is mapped to a vector in ℝ^3^, maintaining consistency with the spatial dimensions of the fMRI data.

#### 2.4.2 Vietoris-Rips filtration and persistence diagram

Persistent homology is then computed from the obtained 3D point clouds to extract topological features. This is done by constructing a series of simplicial complexes using Vietoris-Rips filtration and calculating their homological features. These features capture the underlying topological structure in the data, highlighting meaningful patterns and differences between MCI and its subtypes. We exploit the information encoded in *persistence diagram* to analyze the differences in topology of brain networks of individuals with MCI from HC. A persistence diagram is a graphical representation used in topological data analysis to summarize the topological features (such as connected components, loops, or voids) of a dataset across different scales. It captures the “birth” and “death” of these features as the dataset is gradually transformed or filtered. Formally, a persistence diagram is a multiset of points (b, d), where, “b” (birth) represents the scale at which a topological feature first appears, and “d” (death) represents the scale at which the feature disappears or merges with another feature. The persistence of a topological feature is defined by the difference “d–b,” which measures how long the feature persists over the filtration process. In a persistence diagram, each point corresponds to a topological feature. Features that persist longer (i.e., have a larger “d–b”) are considered more significant. Thus, the persistence diagram encodes the persistence features in data across the filtration parameter range as a collection of points in the two-dimensional Euclidean space ℝ^2^.

A common approach for constructing a filtration from a point cloud is through the Vietoris-Rips complex. This complex is generated from the point cloud by connecting any subset of points whose pairwise distances fall within a specified threshold, creating a simplex. The *Vietoris-Rips filtration* is a method for constructing a sequence of simplicial complexes from a point cloud, where each simplicial complex represents the connectivity structure of the data at a particular scale. It is one of the most commonly used filtrations in topological data analysis. Thus, filtration is a collection $F={\left\{F_{\epsilon }\right\}}_{\epsilon \geq 0}$ of spaces with $F_{\epsilon }\subset F_{\epsilon^{\prime }}$ continuous ∀ϵ ≤ ϵ′. The *i*th persistence diagram of F is a multiset $dgm_{i}\left(F\right)\subset \left\{\left(p,q\right)\in \left[0,\infty \right]\times \left[0,\infty \right]|0\leq p<q\right\}$ where each pair $\left(a,b\right)\in dgm_{i}\left(F\right)$ encodes a *i*-dimensional topological feature, in other words Betti descriptors^2^ associated with a simplicial complex that born at *F*_*b*_ and dies at *F*_*d*_. Here, persistent homology features were computed for 0-dimension, 1-dimension, and 2-dimension separately. The quantity (*d* − *b*) is the persistence of the feature, and typically measures significance across the filtration. In our study, given a time series (*V*_*t*_) the sliding window point cloud ${\mathrm{SW}}_{M,\tau }f$ is computed which is in a metric space (*X, M*_*X*_). The Rips filtration $\mathrm{VR}\left(X,M_{X}\right)$ is derived from the Vietoris—Rips complex *VR*_ϵ_(*X, M*_*X*_), computed at each scale ϵ ≥ 0. The mathematical expression for computing Rips filtration is depicted in Equation 3.


(3) $$\begin{array}{l} \;VR(X,M_{X}):=\{VR_{\epsilon }(X,M_{X}){\}}_{\epsilon \geq 0},where\\ VR_{\epsilon }(X,M_{X}):=\{\{x_{0},...,x_{n}\}\in X|\underset{0\leq i,j\leq n}{\max}M_{X}(x_{i},x_{j})<\epsilon ,n\in \mathbb{N} \end{array}$$

The birth-death pairs (*b, d*) in the Rips persistence diagrams $dgm_{i}^{\mathrm{VR}}\left(X\right):=dgm_{i}\mathrm{VR}\left(X,M_{X}\right)$ reveal the underlying topology of space *X*. The points (*b, d*) in $dgm_{i}^{\mathrm{VR}}\left(X\right)$ with large persistence values (*d* − *b*) suggest the most persistent topological features of the continuous space where *X* is concentrated.

### 2.5 Persistent homology using graph filtration

#### 2.5.1 Graph construction using marginal and partial correlation

A network consists of nodes (vertices) and links (edges) that connect pairs of nodes. In the context of brain networks, the nodes correspond to distinct brain regions, while the edges represent the strength of connectivity between them. Mathematically, this network can be represented as an undirected graph *G* = (*V, E*), where *V* is the set of nodes and *E* is the set of edges. Each edge *l*_*ij*_ ∈ *E*, connecting node *i* with node *j*, has an associated weight—positive or negative—that reflects the temporal correlations in brain activity between the two regions. The entire network is captured in a symmetric adjacency matrix of size *N* × *N*, where *N* is the number of nodes. In this matrix, each entry (*i, j*) indicates the strength of the edge between nodes *i* and *j*. In our study, these edge weights are computed using both marginal and partial correlations.

Correlation analysis has long been a prevalent method for investigating connectivity between brain regions (Kim et al., 2015; Wang et al., 2016). Most studies have relied on Pearson correlation, also known as marginal correlation, which captures only the marginal associations between network nodes. However, using Pearson correlation alone is insufficient for brain connectivity analysis, as it does not account for the true or direct connections between nodes. For example, significant correlations between two nodes, X and Y, may arise due to their shared connection with a third node, Z, even when X and Y are not directly connected (Kim et al., 2015). This reliance on marginal correlation complicates the distinction between network edges representing true connectivity and those influenced by confounding factors. To overcome this limitation, partial correlation has emerged as a powerful statistical technique (Smith, 2012; Kim et al., 2015; Wang et al., 2016). Partial correlation estimates the relationships between nodes while controlling for the spurious effects of all other nodes in the network, providing a more accurate measure of direct connectivity. A zero value in partial correlation indicates an absence of direct connectivity between node pairs. Extensive literature demonstrates that partial correlation is one of the most effective techniques for identifying true functional connectivity between network nodes, often outperforming traditional methods and showing high sensitivity in revealing genuine network connections (Kim et al., 2015; Erb, 2020; Zhang et al., 2021). In this study, we focus exclusively on positively correlated networks for further analysis. Positive networks are constructed when both marginal and partial correlation edge strengths indicate a positive association. The visualization of a positively correlated network for one subject is presented in Figure 1F.

#### 2.5.2 Graph filtration

Consider a weighted graph *G* = (*V, E*), where *V* and *E* represent the sets of vertices and edges, respectively. The weight of an edge *e* is denoted by *w*(*e*). A filter function *f* : *G* → ℝ, on *G*, is defined as follows:

- for an edge *e* ∈ *E*, the value of *f*(*e*) is set to *w*(*e*),
- for a vertex *v* ∈ *V*, the value of *f*(*v*) is obtained by selecting the minimum among all edge weights associated with the edges incident on *v*.

**Figure 3:**
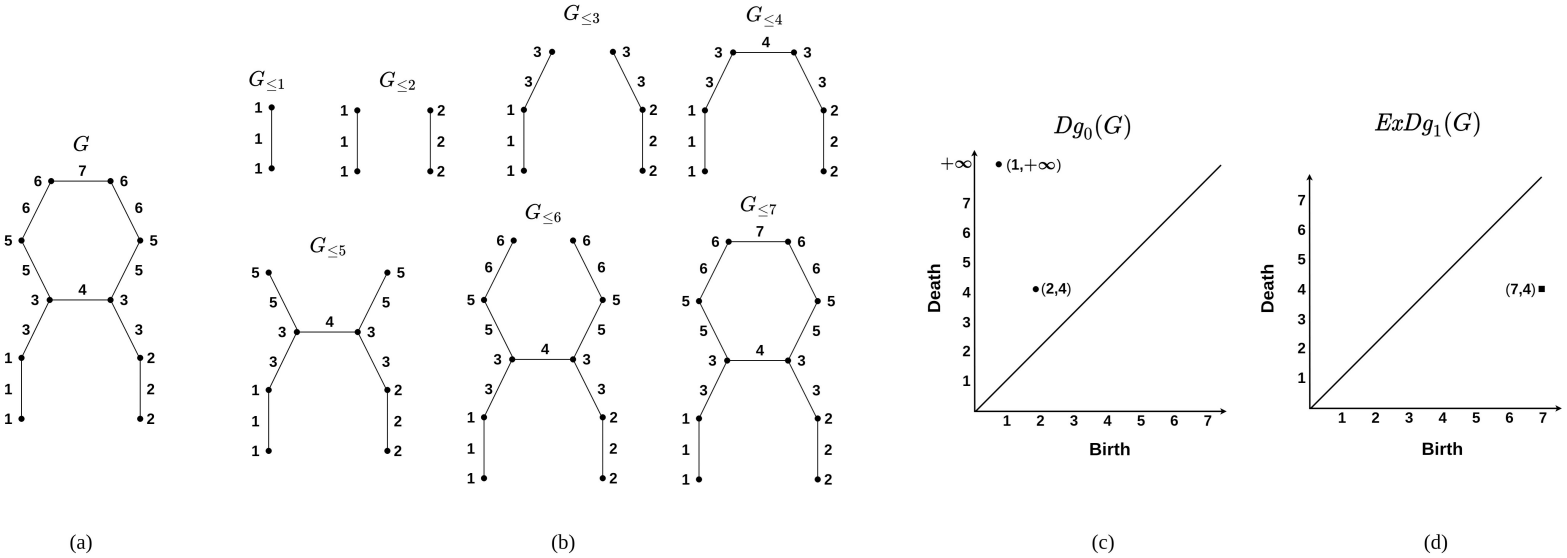
Construction of persistence diagrams from a graph. **(A)** A graph *G*. **(B)** Sublevel set filtration corresponding to *G* based on a filter function *f* : *G* → *R*. The values of *f* are indicated at the vertices and edges. **(C)** 0-th ordinary persistence diagram *Dg*_0_(*G*). Here, A component born at *G*_≤ 2_ merges with another component born at *G*_≤ 1_. This merging event happens at *G*_≤ 4_ and is represented by the point (2, 4). Additionally, the component born at *G*_≤ 1_ never dies, which is indicated by the point (1, ∞). **(D)** 1-st extended persistence diagram *ExDg*_1_(*G*). Here, for a loop *l* in *G*, let 4 and 7 be the minimum and maximum values of *f* along the edges of *l*. The loop is then captured by the point (7, 4) in *ExDg*_1_(*G*). The points in ordinary and extended persistence diagrams are denoted by circular points and squares, respectively.

For each *r* ∈ ℝ, the subgraph *G*_ ≤ *r*_ of *G* is defined as the collection of vertices and edges in *G* with *f*-values at most *r*. Similarly, the subgraph *G*_≥*r*_ consists of vertices and edges with *f*-values greater than or equal to *r*. Let *w*_1_ ≤ *w*_2_ ≤ ⋯ ≤ *w*_|*E*|_ be the weights of the edges in *G*, in non-decreasing order, where |*E*| denotes the number of edges in *G*. This arrangement yields two sequences of graphs, each referred to as a filtration. The sequence ∅ = *G*_≤ 0_ ⊆ *G*_≤_*w*__1__ ⊆ *G*_≤_*w*__2__ ⊆ ⋯ ⊆ *G*_≤_*w*__*n*__ = *G* is called the sublevel set filtration of *f*, and the sequence *G*_≥_*w*__*n*__ ⊆ *G*_≥_*w*__*n*−1__ ⊆ ⋯ ⊆ *G*_≥_*w*__0__ = *G* is referred to as the superlevel set filtration of *f*. To capture the 0-dimensional homological features, one analyzes the birth and death of connected components within the sublevel set filtration. In computational topology, connected components are considered 0-dimensional homological features. Therefore, the persistence diagram that records the birth and death of these components is referred to as the 0-th ordinary persistence diagram, denoted by *Dg*_0_(*G*). To capture loops in a given structure, one examines the birth and death of homology classes within a filtration that integrates both sublevel set and superlevel set filtrations, referred to as the *extended filtration*. Consequently, the persistence diagram that encodes the loops of *G* is known as the 1*-st extended persistence diagram* and is denoted by *ExDg*_1_(*G*). To construct *ExDg*_1_(*G*), one focuses on 1-dimensional homology classes that persist through the sequence of absolute homology groups corresponding to the sublevel set filtration. These homology classes are termed *essential homology classes*. Their deaths are then determined in the relative homology groups associated with the superlevel set filtration. An essential homology class that emerges in the sublevel set filtration and eventually disappears in the superlevel set filtration is represented as a point in *ExDg*_1_(*G*), encoding the topological persistence of loops in the extended filtration framework. The illustration of computing the persistence diagrams using graph filtration is shown in Figure 3. The filtrations provide a basis for computing the persistent topological features that exist within the graph. Here, we compute both *Dg*_0_(*G*) and *ExDg*_1_(*G*) corresponding to each brain networks and utilize them for further analysis.

**Figure 4:**
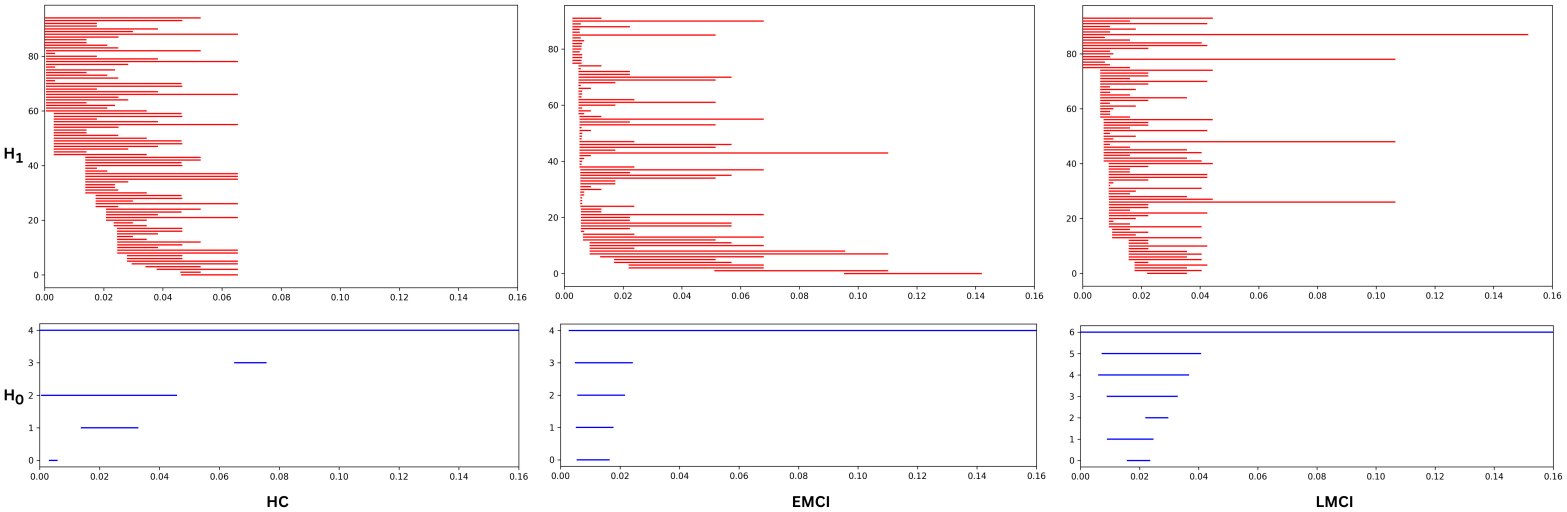
The persistence barcode for a representative subject from the Healthy, EMCI, and LMCI groups for the Occipital (OP) network. The barcode visually represents the birth and death of topological features (connected components *H*_0_ and loops *H*_1_) across different filtration values. Each horizontal bar corresponds to a homological feature, where its length indicates its persistence. Longer bars signify more persistent and structurally significant topological features, while shorter bars represent transient features likely influenced by noise or minor variations in the data. The differences in barcode patterns across groups highlight variations in brain network topology associated with cognitive decline.

Thus, persistence diagrams are generated for all subjects across six distinct brain networks to effectively capture and compare the topological features inherent between each group. This approach provides valuable insights into the structural differences among the groups. A visual representation of these raw topological features is illustrated through persistence barcodes, as shown in Figure 4. This figure presents the persistent homology features for one representative subject from each of the three groups. Each horizontal bar in the barcodes corresponds to a specific topological feature, with the start and end points of each bar indicating the birth and death of that feature, respectively. The length of the bars reflects the persistence of the features; longer bars are interpreted as being more significant, suggesting that these features are more robust and likely to contribute meaningfully to the underlying topology of the network. This visualization allows for an intuitive understanding of the persistence of various topological features and their relevance in distinguishing between the brain networks of the different subject groups.

### 2.6 Quantification of persistence diagrams using Wasserstein distance

To measure the dissimilarity between persistence diagrams, we utilize the Wasserstein distance. This metric is particularly effective in the context of persistence diagrams because it captures not only the differences in the locations of points but also their distribution within the metric space, offering a more comprehensive understanding of the represented topological features (Berwald et al., 2018). In computational topology, the Bottleneck distance and Wasserstein distance are two commonly used metrics for measuring the dissimilarity between two persistence diagrams. Mathematically, this can be expressed as follows:

Suppose, *f*_1_ and *f*_2_ are two different filtrations and let *X* = *dgm*_*p*_(*f*_1_) and *Y* = *dgm*_*p*_(*f*_2_) denote the *p*^*th*^ persistence diagrams corresponding to *f*_1_ and *f*_2_. The Wasserstein and Bottleneck distance metrices are used to quantify the dissimilarity between these two multisets *X* and *Y*. Let *L*_∞_(*f*_1_, *f*_2_) = ∥*f*_1_ − *f*_2_∥_∞_ denote the supremum distance between *f*_1_ and *f*_2_, and η denotes a bijection of *X*→*Y*, then, the *q*−Wasserstein distance between two persistence diagrams *X* and *Y* is defined as


(4) $$\begin{array}{l} W_{q,p}\left(X,Y\right)={\left[\underset{\eta :X\to Y}{\inf}\underset{x\in X}{\sum }∥x-\eta \left(x\right){∥}_{\infty }^{q}\right]}^{\frac{1}{q}} \end{array}$$

To compute the distance elements of *X* and *Y* one-to-one (bijection η) are matched. It is usually done in the following way: first for each pair of elements, *x* ∈ *X* and *y* = η(*x*) ∈ *Y*, the difference between them (the cost function) is calculated using ||*x* − η(*x*)||_∞_ that is basically *L*_∞_ norm. Adding up the *q*th degrees $∥.{∥}_{\infty }^{q}$, we get a notion of the difference between the whole multisets X and Y under the matching η : *X* → *Y*. Taking the infimum over all possible bijections η, we get the difference between multisets *X* and *Y* under the best matching possible, effectively removing η from further consideration. The bottleneck distance is the Wasserstein distance, with parameter *q* → ∞. Hence, one drawback of the bottleneck distance is its insensitivity to details of the bijection beyond the furthest pair of corresponding points, which can result in a loss of important information. Due to this, the present study considers Wasserstein distance for quantification. A low value of Wasserstein distance suggests that the two fMRI time series show similar patterns of neural activity over time, indicating that the two brain regions are functionally synchronized and likely involved in coordinated activity. In contrast, a high value of Wasserstein distance indicates that the fMRI time series of the two brain regions have distinct patterns of neural activity. This may imply that the regions are functionally dissociated or independent. High Wasserstein distances could also point to abnormalities in functional connectivity between the regions, potentially signaling neurological or psychiatric disorders.

#### 2.6.1 *Network-specific* inter-subject Wasserstein distance

Network-specific inter-subject Wasserstein distance was computed for the two Betti descriptors: *H*_0_ and *H*_1_, generated using graph filtration. The Wasserstein distance between the persistence diagrams of the positively correlated connectivity graphs corresponding to subjects *S*_1_ and *S*_2_, quantifies the structural similarity in the topology of their brain networks. A low Wasserstein distance indicates that the subjects *S*_1_ and *S*_2_ have similar topological structures in their brain networks, suggesting functional synchrony or comparable activity between their brain regions. Conversely, a high Wasserstein distance signifies greater topological differences between the subjects' brain networks, implying distinct connectivity patterns or functional dissociation. This difference may point to abnormalities in brain connectivity, potentially associated with neurological or psychiatric conditions. In the context of the three groups (HC, EMCI, and LMCI), analyzing these distances across brain networks such as CB, CO, DMN, FP, OP, and SM helps to reveal MCI specific differences in brain network organization. To analyze differences in topological patterns across all subjects from the three groups (HC, EMCI, and LMCI) for each of the two Betti descriptors (*H*_0_ and *H*_1_), a network-specific analysis is performed for the six brain networks (CB, CO, DMN, FP, OP, SM). This is illustrated by the Wasserstein distance matrix (*WD*_*S*_), which has dimensions *N* × *N*, where *N* represents the number of subjects. Equation 5 provides the mathematical representation of the *WD*_*S*_ matrix. In this matrix, each element *WD*_*S*_(i, j) reflects the Wasserstein distance between the persistence diagrams of subject i and subject j for a particular network.


(5) $$\begin{array}{l} WD_{S}\\ =\left[\begin{array}{ccc} W_{q,p}\left(PD\left(Subj_{1}\right),PD\left(Subj_{1}\right)\right) & \ldots & W_{q,p}\left(PD\left(Subj_{1}\right),PD\left(Subj_{N}\right)\right)\\ W_{q,p}\left(PD\left(Subj_{2}\right),PD\left(Subj_{1}\right)\right) & \ldots & W_{q,p}\left(PD\left(Subj_{2}\right),PD\left(Subj_{N}\right)\right)\\ \vdots & \ddots & \vdots \\ W_{q,p}\left(PD\left(Subj_{N}\right),PD\left(Subj_{1}\right)\right) & \ldots & W_{q,p}\left(PD\left(Subj_{N}\right),PD\left(Subj_{N}\right)\right) \end{array}\right] \end{array}$$

A sample image as obtained from *WD*_*S*_ for one specific network is shown in Figure 1H.

#### 2.6.2 *Subject-specific* inter-ROI Wasserstein distance

For each brain network, subject-specific inter-ROI Wasserstein distance was computed for each of the three Betti descriptors: *H*_0_, *H*_1_, and *H*_2_, generated using Vietoris-Rips filtration. The Wasserstein distance between fMRI time series from two brain regions reflects the similarity in their neural activity patterns. A low Wasserstein distance suggests that the two fMRI time series share similar activity patterns over time, indicating that the regions are functionally synchronized and likely engaged in coordinated activity. Conversely, a high Wasserstein distance implies distinct neural activity patterns between the two regions, which may be functionally dissociated or independent. Additionally, high Wasserstein distances could signal abnormalities in functional connectivity between the regions, potentially pointing to neurological or psychiatric disorders. The pairwise-ROI distance matrix (*WD*_*ROI*_), with dimensions *n* × *n*, where *n* denotes the number of regions of interest (ROIs) in a specific brain network, captures the interaction between different ROIs. Each entry in the matrix *PR*(*i, j*) represents the Wasserstein distance between the persistence diagrams of ROI *i* and ROI *j*. The mathematical representation of the matrix *WD*_*ROI*_ is shown in Equation 6. Calculating both the persistent homology and the Wasserstein distance matrices (PR and PS) is computationally demanding. To address this challenge, we employed high-performance computing (HPC) resources, specifically an Intel(R) Xeon(R) Gold 6240 CPU @ 2.60GHz with dual CPUs and 192 GB of memory.


(6) $$\begin{array}{l} WD_{ROI}\\ =\left[\begin{array}{ccc} W_{q,p}\left(PD\left(ROI_{1}\right),PD\left(ROI_{1}\right)\right) & \ldots & W_{q,p}\left(PD\left(ROI_{1}\right),PD\left(ROI_{n}\right)\right)\\ W_{q,p}\left(PD\left(ROI_{2}\right),PD\left(ROI_{1}\right)\right) & \ldots & W_{q,p}\left(PD\left(ROI_{2}\right),PD\left(ROI_{n}\right)\right)\\ \vdots & \ddots & \vdots \\ W_{q,p}\left(PD\left(ROI_{n}\right),PD\left(ROI_{1}\right)\right) & \ldots & W_{q,p}\left(PD\left(ROI_{n}\right),PD\left(ROI_{n}\right)\right) \end{array}\right] \end{array}$$

Figure 1E illustrates the variability in Wasserstein distance among all ROI pairs for a single representative subject.

### 2.7 Classification

Classification is conducted using two distinct sets of features:

*Set-1:* This set comprises raw features derived from the persistence diagrams, specifically focusing on the lifespan of topological descriptors calculated using graph filtration. These features provide insights into the persistence and significance of various topological structures within the brain networks.

*Set-2:* This set includes the inter-region of interest (ROI) Wasserstein distances computed using Vietoris-Rips filtration. These distances quantify the dissimilarities between the persistence diagrams of different brain regions, capturing essential topological information about connectivity patterns.

For classification, we employ a stacked ensemble classifier for the first set of features, leveraging its ability to combine multiple learning algorithms to improve predictive performance. In contrast, we utilize a custom convolutional neural network (CNN) model for the second set of features, which is designed to effectively capture spatial hierarchies and complex patterns within the Wasserstein distances. This dual approach allows us to maximize the strengths of both feature sets, enhancing our overall classification accuracy.

#### 2.7.1 Stacked ensemble classifier

An ensemble classifier integrates the predictions from multiple models to achieve a more accurate and robust classification than any individual model could provide. By leveraging the strengths of various algorithms, this approach reduces the likelihood of errors and enhances the model's ability to generalize to new data. In our case, the ensemble method was employed to capitalize on the complementary advantages offered by different classifiers. The classification process is based on the top ten most persistent homology features, where the lifespan of each feature in *H*_0_ and *H*_1_ serves as the primary input for training. The dataset is divided into training (80%) and testing (20%) subsets, ensuring that the model's performance could be assessed on unseen data after training, thereby providing a realistic evaluation of its generalization capabilities. First, feature selection is performed using Recursive Feature Elimination (RFE), a technique that systematically removes the least important features to identify the most relevant ones. A Random Forest classifier was utilized within RFE to rank and eliminate features, ultimately pinpointing the five most significant features from the original ten persistent homology points. Once the key features are selected, a stacking ensemble was constructed, incorporating a diverse array of base classifiers, including Support Vector Classifier, Random Forest, Gradient Boosting, XGBoost, AdaBoost, Extra Trees, Logistic Regression, K-Nearest Neighbors, LightGBM, and CatBoost. Each of these models was trained independently on the training dataset, allowing them to capture different patterns and relationships within the data that a single model might overlook. To ensure a robust assessment of model performance, all base models were evaluated using stratified K-Fold cross-validation (*K* = 5). This technique guarantees that each fold maintains the same proportion of samples from each class, effectively addressing potential issues with imbalanced data and providing a more reliable estimate of model accuracy. Following this, the classifiers were ranked based on their cross-validation accuracy scores, and the top-performing models were selected for inclusion in the final stacking ensemble. The stacking classifier was assembled using the five highest-ranked models, with a Random forest classifier designated as the meta-model to aggregate the predictions from the base classifiers. The predictions from these five top models on the training dataset were then used as inputs for the meta-classifier, allowing the stacking approach to leverage the unique strengths of each individual classifier, thereby further enhancing overall performance. The model was trained across four scenarios: (1) HC vs. EMCI; (2) HC vs. LMCI; (3) EMCI vs. LMCI for the ADNI dataset; and (4) HC vs. MCI for the in-house TLSA dataset. Finally, the performance of the model is tested on unseen data. This comprehensive approach ensures that our classification model is both robust and effective in differentiating between various cognitive states.

#### 2.7.2 Custom CNN

**Figure 5:**
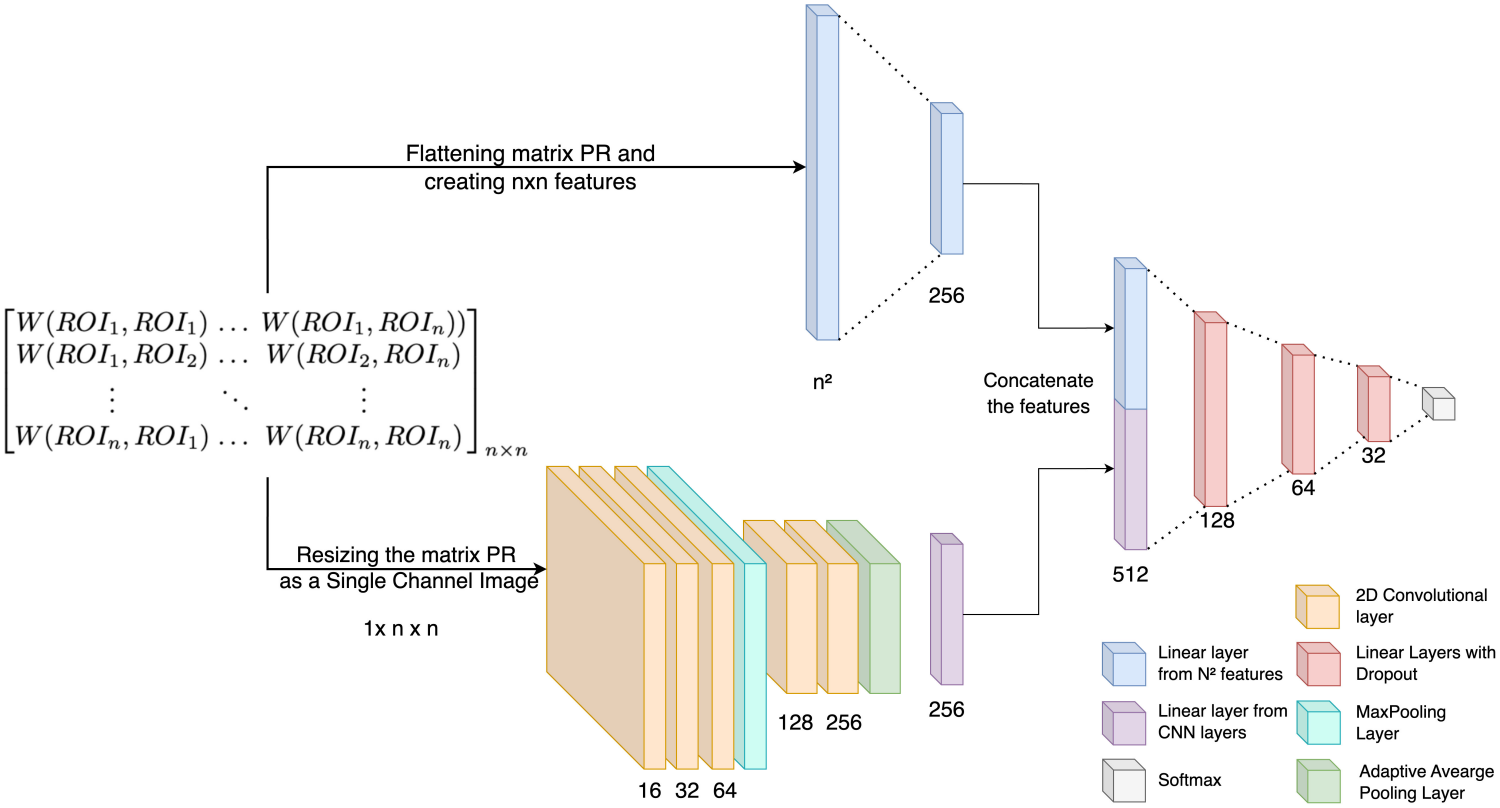
The deep learning architecture for classification using persistent diagrams generated with Vietoris-Rips filtration.

The study incorporates both 1D and 2D features derived from Wasserstein distances, which capture inter-ROI interactions for each subject, into a classification framework utilizing a conventional CNN. For classification, a subject-specific *WD*_*ROI*_ matrix (*n* × *n*) is employed. The proposed CNN architecture, illustrated in Figure 5, combines 1D features extracted from each ROI pair in the *WD*_*ROI*_ matrix with 2D CNN-derived features. The proposed classification model operates in two steps. First, the Wasserstein distance matrix is flattened to generate 1D features, focusing on pairwise relationships. Then, 2D features are extracted from the matrix using CNN layers, capturing local patterns and spatial hierarchies (Figure 1E). These features are concatenated to form a unified feature vector, integrating information from both linear and convolutional layers. This unified vector is processed through several dense layers with dropout for regularization, minimizing the risk of overfitting. The combination of 1D and 2D features results in a richer, more diverse feature space that captures various aspects of the data—1D features represent sequential or linear relationships, while 2D features capture spatial or topological relationships. This integration offers multiple benefits, including a comprehensive feature space for classification, improved learning from different perspectives, noise and artifact mitigation, and the ability to capture both local and global patterns. For the 2D features, the model includes three CNN layers with 16, 32, and 64 filters, followed by a max-pooling layer and an additional convolutional block with two CNN layers containing 128 and 256 filters. All CNN layers use a kernel size of 3. A global average pooling layer then condenses each feature map into a single value, forming a linear feature vector, with ReLU activation functions applied throughout. Simultaneously, the 1D features of the *WD*_*ROI*_ matrix (*n*^2^ × 1) are processed through a linear layer, reducing them to 256 features. The 256-dimensional feature vectors from both the linear layer and the 2D CNN are concatenated and passed through a series of fully connected layers with sizes 128, 64, and 32, each incorporating a dropout rate of 0.2. The final layer employs a softmax activation function for classification. Each Betti descriptor (*H*_0_, *H*_1_, and *H*_2_) from every brain network is independently analyzed. We used Optuna for hyperparameter tuning within the framework of cross-validation. Optuna was employed to optimize the hyperparameters for each fold of the cross-validation process. For each fold, the best learning rate, batch size, train-test split ratio, and optimizer type were selected. This approach ensured that the model was tuned and evaluated across multiple folds, providing a more reliable estimate of its performance. Specifically, 20 trials were performed to determine the best learning rate, which was chosen from the range 10^−4^ to 10^−1^. The batch size was selected from the options 4, 8, 16, 32, and the train-test split ratio was chosen from 0.2, 0.25, 0.3, based on unique subject IDs. The optimizer was chosen between SGD and Adam. Additionally, early stopping with a patience of 10 was implemented, with the initial number of epochs set to 100 to prevent overfitting. Cross-Entropy loss is used for training. The classification tasks include (i) MCI vs. HC (for both ADNI and our in-house dataset), (ii) EMCI vs. HC (ADNI), (iii) LMCI vs. HC (ADNI), and (iv) EMCI vs. LMCI (ADNI). The entire experiment is conducted on 24 GB NVIDIA A5000 GPUs and the PyTorch deep learning framework.

## 3 Results

This study utilizes persistent homology to investigate variations in brain network topology between healthy individuals and those diagnosed with MCI having different stages (Early/Late). Positively correlated graphs are generated for six classical brain networks—CB, CO, DMN, FP, OP, and SM—derived from 160 Dosenbach ROIs, shown in Figure 2. These brain connectivity graphs are constructed based on the rs-fMRI time series of each network, considering only instances where both marginal and partial correlations exhibit positive values.

First, graph filtration is employed to compute persistent homology for dimension-0 and -1 across each of the six brain network. The resulting persistence diagrams are then examined to identify key topological features. As outlined in the methodology, each point in a persistence diagram represents a specific topological feature, where the difference between the “birth” and “death” values signifies the lifespan or persistence of a feature. Longer persistence indicates more prominent or stable topological characteristics. Figure 4 visually illustrates significant differences in topological features among the three groups: HC, EMCI, and LMCI. The persistence barcodes for one representative subject from each group are shown for both dimension-0 (connected components) and dimension-1 (loops). The control group exhibits fewer topological features, and these features tend to have shorter lifespans, indicating more transient or less complex network structures. Additionally, there is minimal variation in the persistence of these features, suggesting more uniform brain network topology across healthy individuals. In contrast, the EMCI group displays a wider range of topological features. While some features have short lifespans, similar to those in the HC group, EMCI also shows several features that persist for longer duration. This suggests that EMCI individuals have more complex and diverse topological structures than HC, potentially reflecting early-stage disruptions in brain network connectivity. The LMCI group, on the other hand, presents a distinctive pattern. Most features in the persistence diagrams have relatively short lifespans, indicating that the majority of topological structures in their brain networks are transient or unstable. However, LMCI also demonstrates a few long-persisting features, which last longer than those observed in both the HC and EMCI groups. These features may represent more pronounced or severe alterations in brain connectivity, characteristic of late-stage MCI.

### 3.1 Significant topological differences are obtained in raw persistence features between HC and MCI-subtypes

As discussed in Section 2.6.1, inter-subject Wasserstein distances (shown in Figure 1H) are computed from the raw persistence features derived through graph filtration to assess dissimilarities between the persistence diagrams of healthy and diseased groups. In this study, the Wasserstein distance serves as a metric for comparing the raw persistent homology features of brain connectivity graphs, providing valuable insights into structural variations across different subjects. In order to determine whether the topological features as obtained through persistent homology differ significantly between study groups, we tested the hypothesis that the difference in mean distribution of Wasserstein distances is statistically significant between healthy individuals and MCI groups. For this, the Wilcoxon rank-sum test is conducted at a 95% confidence interval for each of the six brain networks. Table 2 presents the statistical results for the inter-subject Wasserstein distance computed from the 0- and 1-dimensional raw persistent homology features for both the ADNI and TLSA datasets. The results show statistically significant differences (*p* < 0.001) in most persistence features across all six networks between healthy and MCI groups, as well as between different MCI sub-types.

**Table 2:** Result of Wilcoxon rank-sum test at 95% C.I. to check if the changes in Wasserstein distance is statistically significant.

**Network**	**Homology**	**ADNI data**			**TLSA data**
	**Dimension**	**HC vs. EMCI**	**HC vs. LMCI**	**EMCI vs. LMCI**	**HC vs. MCI**
CB	*H*_0_	*p* < 0.001	*p* < 0.001	*p* < 0.001	*p* < 0.001
	*H*_1_	*p* < 0.001	*p* < 0.001	NS	*p* < 0.001
CO	*H*_0_	*p* < 0.001	*p* < 0.001	*p* < 0.001	NS
	*H*_1_	*p* = 0.01	NS	NS	*p* < 0.001
DMN	*H*_0_	*p* < 0.001	*p* < 0.001	*p* < 0.001	*p* < 0.001
	*H*_1_	*p* < 0.001	*p* < 0.001	*p* < 0.001	*p* = 0.03
FP	*H*_0_	*p* = 0.02	*p* < 0.001	*p* < 0.001	*p* < 0.001
	*H*_1_	*p* < 0.001	*p* < 0.001	*p* < 0.001	NS
OP	*H*_0_	*p* < 0.001	*p* = 0.001	*p* < 0.001	*p* < 0.001
	*H*_1_	*p* = 0.001	*p* < 0.001	*p* < 0.001	*p* < 0.001
SM	*H*_0_	NS	*p* < 0.001	*p* < 0.001	*p* = 0.008
	*H*_1_	*p* < 0.001	NS	*p* < 0.001	NS

This is performed using top 10 most persistent features, between Healthy Control (HC), early MCI (EMCI), and late MCI (LMCI) for different brain networks in ADNI data and TLSA data.

### 3.2 Classification using top ten most persistent features

The analysis of persistence diagrams reveals notable differences in the topological characteristics of MCI and its sub-types compared to HC, as derived through graph filtration. The differences in the number, persistence, and variation of topological features provide valuable insights into the progression of brain network changes from healthy to early and late MCI. As discussed earlier, features that persist longer tend to better capture the underlying topological structure of the graph compared to the features that do not persist longer and might actually be a result of noise. Therefore, for classification purpose, the top ten most persistent features of *H*_0_ and *H*_1_ are used separately to differentiate the three groups using stacked ensemble classifier. However, while comparing the classification performance between the two homology dimensions, the best classification accuracy is found with most persistent *H*_1_ features, with 63.9% in classifying EMCI vs. LMCI in the DMN. For our in-house TLSA data, the model classified HC vs. MCI with the accuracy of 71.4% in the DMN. The performance of the stacked classifier in classifying MCI sub-types and healthy controls is summarized in Table 3. While graph filtration successfully captures some degree of network topology, the model's limited performance suggests that the raw persistent homology features may not fully exploit the complexity of the underlying brain network's structure, particularly in more challenging classification tasks.

**Table 3:** The classification accuracy (in %) as obtained across six functional brain networks using stacked ensemble classifier with top ten most persistent raw homology features for dimension-1.

**Network**	**ADNI data**			**TLSA Data**
	**HC vs. EMCI**	**HC vs. LMCI**	**EMCI vs. LMCI**	**HC vs. MCI**
DMN	53.6	50.0	**63.9**	**71.4**
FP	55.1	51.6	50.8	60.0
OP	57.9	**56.0**	49.0	50.0
SM	53.6	54.7	57.0	66.67
CO	**61.8**	53.0	54.0	60.0
CB	58.8	50.0	59.0	60.9

The maximum accuracy values, obtained from graph filtration are shown in “bold”.

### 3.3 Classification using inter-ROI Wasserstein distance as features

In addition to graph filtration, Vietoris-Rips filtration is utilized to derive persistence diagrams. As previously discussed, Vietoris-Rips filtration applies persistent homology to dimensions 0, 1, and 2 on 3D point clouds constructed from rs-fMRI time series data. This process helps identify persistent topological features within the point clouds. To quantify the dissimilarities between persistence diagrams, the Wasserstein distance metric is employed. The inter-ROI Wasserstein distance (*WD*_*ROI*_) is calculated for each homology dimension (*H*_0_, *H*_1_, and *H*_2_) based on the persistence diagrams, and these distances are then used as input features in a convolutional neural network (CNN) model for classification. The performance of the proposed CNN model, using inter-ROI Wasserstein distances as features, in classifying MCI and its subtypes is summarized in Table 4. The CNN model achieves a classification accuracy of 85.7% for the in-house TLSA MCI cohort. However, when classifying MCI subtypes from healthy controls, the accuracy drops to 70.8% in distinguishing EMCI from HC and classification of LMCI from HC achieves an accuracy of 81.0%, which is on par with the TLSA HC vs. MCI classification. When distinguishing between EMCI and LMCI, the model attains an accuracy of 77.3%.

**Table 4:** The classification accuracy (in %) as obtained across six distinct brain networks using CNN with Inter-ROI Wasserstein Distance as features.

**Comparison**	**Dataset**	**Homology dimension**	**DMN**	**FP**	**OP**	**SM**	**CO**	**CB**
HC vs. EMCI	ADNI	*H*_0_	**70.8**	56.0	56.7	57.8	61.4	67.2
		*H*_1_	70.2	56.3	66.0	66.7	61.9	60.9
		*H*_2_	66.6	60.0	69.1	66.52	55.4	66.1
HC vs. LMCI	ADNI	*H*_0_	66.7	71.0	**81.0**	60.0	66.1	63.2
		*H*_1_	67.6	58.7	75.0	67.2	68.2	64.3
		*H*_2_	74.6	59.7	65.6	63.6	71.4	50.0
EMCI vs. LMCI	ADNI	*H*_0_	69.8	54.5	68.2	69.9	62.9	63.3
		*H*_1_	58.1	75.4	65.7	70.0	**77.3**	58.3
		*H*_2_	58.7	71.9	57.7	66.7	61.4	71.6
HC vs. MCI	In-house TLSA	*H*_0_	**85.7**	78.6	64.7	78.6	71.4	70.6
		*H*_1_	64.7	64.3	71.4	71.4	82.4	78.6
		*H*_2_	**85.7**	64.7	71.4	78.6	**85.7**	64.3

Split based on Unique subjects. The highest accuracy values are shown in “bold”.

### 3.4 Comparison between graph vs. Vietoris-Rips filtration

**Figure 6:**
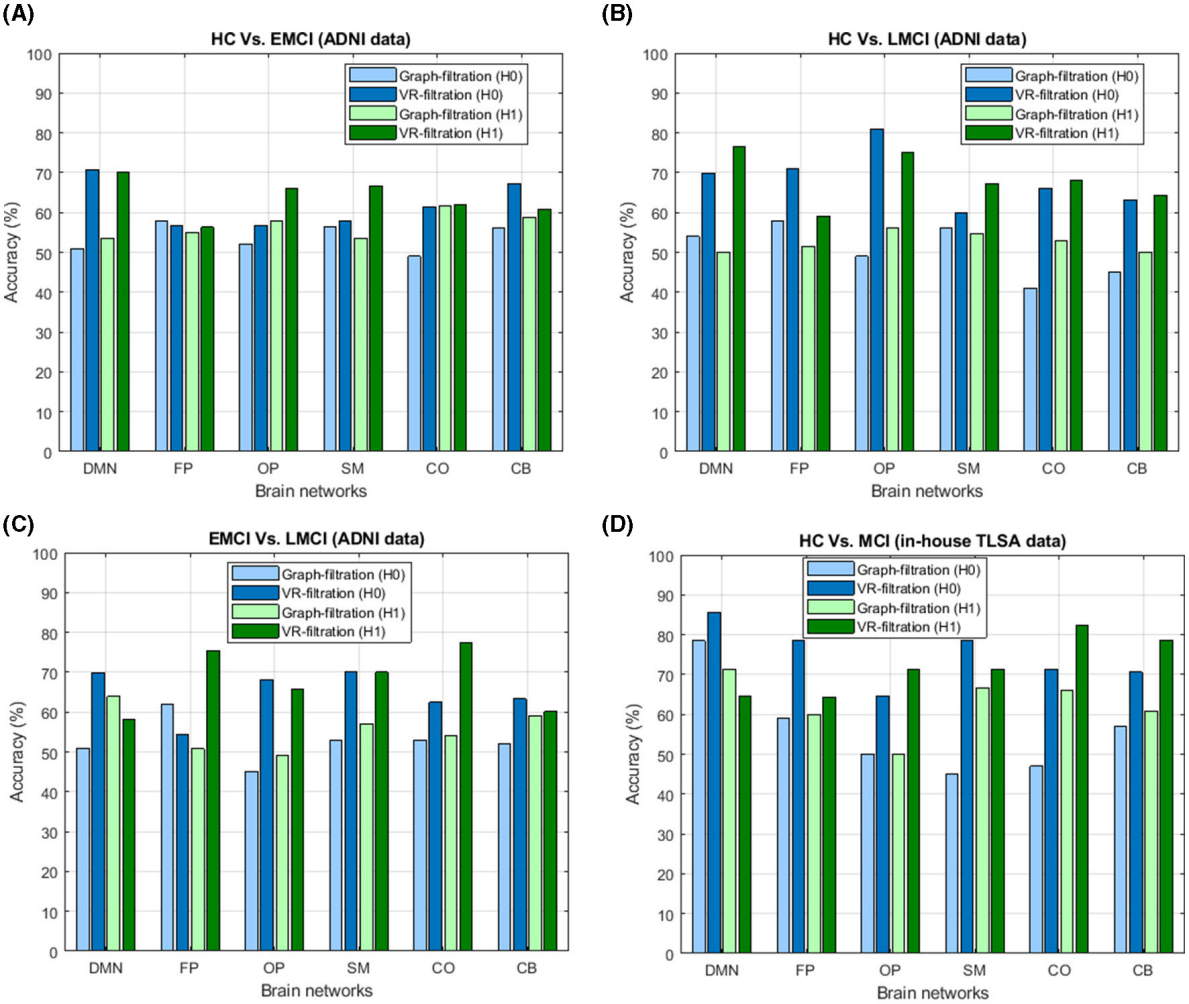
Illustration of the comparative analysis between graph filtration and Vietoris-Rips filtration for both dimension-0 (*H*_0_) and dimension-1 (*H*_1_) in the classification of **(A)** healthy controls (HC) vs. early mild cognitive impairment (EMCI), **(B)** HC vs. late mild cognitive impairment (LMCI), **(C)** EMCI vs. LMCI, and **(D)** HC vs. mild cognitive impairment (MCI) based on TLSA data. The plot clearly highlights the superiority of Vietoris-Rips filtration over graph filtration, showcasing its enhanced ability to differentiate between these cognitive states.

While comparing the classification performance between these two types of filtrations, it is seen that the proposed CNN model using Vietoris-Rips filtration and inter-ROI Wasserstein distance features outperformed the stacked ensemble classifier based on graph filtration in all classification tasks. The Vietoris-Rips filtration demonstrated better accuracy in differentiating between MCI subtypes and HC, indicating that it captures more detailed topological features of brain connectivity networks. This suggests that Vietoris-Rips filtration provides a more robust representation of brain network topology for MCI classification. The superior performance of Vietoris-Rips filtration when comparing these two filtration methods can be attributed to three key factors, which have both methodological and biological significance. This comparison is depicted in Figure 6 through a bar diagram and is further elaborated in the subsequent subsections.

#### 3.4.1 Point cloud representation vs. fixed graph structure

Vietoris-Rips filtration operates on 3D point clouds generated from rs-fMRI time series data, offering a more dynamic and flexible representation of brain connectivity. These point clouds allow the method to capture the underlying topological features of functional connectivity across brain regions. Each point represents temporal interactions between regions, making this method particularly sensitive to the non-linear and time-varying nature of brain dynamics. Graph filtration, by contrast, relies on fixed connectivity matrices, which are based on static, pairwise relationships. While this approach captures local connectivity between specific brain regions, it lacks the flexibility to reflect the dynamic changes that occur over time in the brain's functional network. In neurodegenerative diseases like MCI, brain networks undergo dynamic reorganization as cognitive decline progresses. The flexibility of Vietoris-Rips filtration allows for the detection of subtle changes in functional connectivity over time, offering a deeper understanding of early disease progression. Capturing these temporal variations is crucial for early diagnosis and intervention in MCI, as it provides a more complete picture of how functional networks are disrupted.

#### 3.4.2 Inter-ROI Wasserstein distance vs. lifespan features

The key distinction between the two approaches lies in the use of inter-ROI Wasserstein distance in Vietoris-Rips filtration. This distance metric quantifies the similarity between persistence diagrams across all ROIs, enabling a more direct and comprehensive comparison between subjects. This offers more global insights into how the overall network topology changes in response to disease progression. In contrast, graph filtration uses the lifespan of the topological features (connected components in dimension 0 and loops in dimension 1) within the connectivity matrix. This approach focuses on local features, emphasizing persistence within individual brain regions but potentially overlooking global interactions across different parts of the brain. Neurodegenerative diseases like MCI are characterized by disruptions in inter-regional brain communication and synchronization. The Wasserstein distance, by comparing persistence diagrams across ROIs, better captures the overall dissimilarity in brain network topology between healthy and diseased states. It reflects how disease-related changes impact the global organization of brain networks, including how different regions fall out of synchrony or lose coordination, a hallmark of MCI. In contrast, the lifespan features from graph filtration may miss these more global and inter-regional shifts in connectivity.

#### 3.4.3 Richer topological features in Vietoris-Rips filtration

While both graph filtration and Vietoris-Rips capture topological features like connected components (dimension 0) and loops (dimension 1), Vietoris-Rips goes beyond by incorporating dimension 2 (voids), which graph filtration inherently cannot do due to its 1D simplicial complex structure. This additional dimensionality allows for a more comprehensive representation of brain connectivity, capturing complex relationships among brain regions that could reflect higher-order cognitive processes. Study of this higher-order dynamics could be essential in understanding the breakdown of brain networks in MCI. Since cognitive decline affects complex networks, Vietoris-Rips filtration is better suited to reflect the disruptions in these interactions. Although individual inter-ROI Wasserstein distance features from each dimension are fed into the CNN, the additional dimensionality (*H*_2_) provided by Vietoris-Rips filtration enriches the feature set by capturing higher-order interactions, global topological structures, and interdependencies between dimensions. These factors contribute to the improved classification performance, as they offer a more comprehensive representation of the brain's connectivity network, critical for detecting early pathological changes in MCI. The methodological advantages of Vietoris-Rips filtration, including its capacity to operate with flexible point clouds, the application of inter-ROI Wasserstein distance for global comparisons, and the incorporation of higher-dimensional simplices, hold substantial clinical significance for enhancing our understanding of MCI. The superior performance of Vietoris-Rips filtration in distinguishing MCI sub-types from healthy indivisuals demonstrates its potential to capture complex, dynamic changes in brain connectivity that are crucial for early detection and classification of neurodegenerative diseases. These factors combine to make Vietoris-Rips filtration a more biologically meaningful and technically robust approach for classifying brain network alterations in MCI, offering deeper insights into the disease's impact on brain topology and potential for improved diagnosis and intervention strategies.

### 3.5 Comparison with state-of-the-art techniques

To assess the effectiveness of our proposed methodology, we compared the classification results from our approach with those from previous studies. Various methodologies previously have been employed for the automated diagnosis of MCI sub-types and HC, including the sub-network kernel method (Jie et al., 2018b), a multiple-BFN-based 3D CNN framework (Kam et al., 2020, 2018), integrating temporal and spatial properties of network (Jie et al., 2018a), the Spatial-Temporal convolutional-recurrent neural Network (STNet) (Wang et al., 2020). Although these methods demonstrate commendable performance, our approach of leveraging inter-ROI Wasserstein distance as features for classification, significantly outperforms them. We achieved accuracy of 70.8% for distinguishing between HC and EMCI, 81% for HC vs. LMCI, 77.3% for differentiating between EMCI and LMCI, and 85.7% for HC vs. MCI in the TLSA dataset. Our results specifically highlight the potential of using inter-ROI Wasserstein distance to capture subtle differences in brain connectivity patterns. Table 5 presents a detailed comparison of the classification performance between the proposed methodology and other state-of-the-art approaches, underscoring the robustness and clinical relevance of our findings in the context of MCI diagnosis.

**Table 5:** Comparative analysis of the proposed method with recent state-of-the-art techniques that used fMRI data to differentiate MCI sub-types and healthy.

**References**	**Modality**	**Comparison**	**Accurcay**
Kam et al. (2018)	fMRI (ADNI)	HC vs. EMCI	74.23%
Jie et al. (2018b)	fMRI (ADNI)	EMCI vs. LMCI	74.8%
		HC vs. MCI	82.6%
Jie et al. (2018a)	fMRI (ADNI)	EMCI vs. LMCI	78.8%
Kam et al. (2020)	fMRI (ADNI)	HC vs. EMCI	76.07%
Wang et al. (2020)	fMRI (ADNI)	EMCI vs. LMCI	79.36%
Lee et al. (2021)	fMRI (ADNI)	HC vs. EMCI	74.42%
Yang et al. (2021)	fMRI (ADNI)	HC vs. LMCI	87.23%
Bolla et al. (2023)	fMRI (ADNI)	HC vs. MCI	90%
Ammu et al. (2024)	fMRI (ADNI)	HC vs. MCI	89.47%
**Proposed Methodology (2024)**			
Graph Filtration	fMRI (ADNI)	HC vs. EMCI	61.8%
		HC vs. LMCI	56.0%
		EMCI vs. LMCI	63.9%
	In-House TLSA	HC vs. MCI	71.4%
Vietoris-Rips Filtration	fMRI (ADNI)	HC vs. EMCI	**70.8%**
		HC vs. LMCI	**81.0%**
		EMCI vs. LMCI	**77.3%**
	In-House TLSA	HC vs. MCI	**85.7%**

The best result as obtained from the proposed methodology is shown in “bold”.

## 4 Discussion

Over the past two decades, numerous fMRI studies have been conducted to examine brain connectivity patterns in individuals with neurological and psychiatric disorders, as well as in healthy controls, both during various cognitive tasks and in resting-state conditions (Bhattacharya et al., 2025; Devika and Ramana Murthy, 2021; Devisetty and Amsitha, 2022; Venkatapathy, 2023; Manickam et al., 2024). However, the lack of a standardized clinical test and the absence of a cure for dementia have prompted the exploration of machine learning as a means to identify individuals at risk of developing cognitive impairment, thereby enabling proactive intervention (Stamate et al., 2018). In this context, the application of machine learning techniques has shown significant promise, with studies reporting their ability to accurately differentiate between individuals with early and late stages of MCI, as well as those who are cognitively healthy (Stamate et al., 2018; Danso et al., 2021; Basheera and Sai Ram, 2019). One of the key advantages of using machine learning is its capacity to identify subtle, complex patterns in data that may not be easily discernible to the human eye. This is particularly relevant in the context of neurodegenerative diseases, where the underlying pathological changes often occur years before the onset of clinical symptoms (Danso et al., 2021).

Numerous studies have attempted to distinguish MCI and its sub-types, often with moderate success in terms of classification accuracy. Unlike traditional methods, our approach introduces an innovative technique grounded in computational topology, specifically utilizing persistent homology from topological data analysis. The proposed study is unique in its application of machine learning combined with persistent homology to explore changes in brain network topology between HC and MCI subtypes (early and late) using fMRI time series data. While most state-of-the-art techniques, such as deep learning and network-based methods, focus on spatial and temporal features or rely on predefined connectivity metrics, our approach captures deeper, more intrinsic properties of brain networks. Persistent homology allows us to identify topological structures, such as connected components, loops, and voids, offering a global view of brain connectivity beyond pairwise interactions. Our study goes beyond conventional network metrics by applying topological data analysis to brain networks, enabling a deeper exploration of the complex geometry and topology of brain function. This approach captures subtle structural differences in brain network topology that are often overlooked by traditional methods. Additionally, Persistent homology is leveraged using two different kinds of filtration techniques: Vietoris-Rips filtration on 3D point cloud and graph filtration on positively correlated network, as constructed from rs-fMRI time series data. This dual approach introduces novelty by offering a more comprehensive understanding of brain network topology. The graph filtration captures local connectivity features, while Vietoris-Rips filtration incorporates higher-dimensional interactions and global topological insights. By comparing these methods, the study highlights how different filtration techniques can reveal unique aspects of brain network reorganization in MCI; setting a new direction for the application of topological data analysis in neurological research.

In case of Vietoris-Rips filtration, the 3D point cloud is generated from 1D fMRI time series using sliding window embedding. Vietoris-Rips filtration is then applied to construct simplicial complexes and compute persistent homology features across dimension-0, 1, and 2. Inter-ROI Wasserstein distances are computed between persistence diagrams for each subject and for each dimension which is then used as features for classification via a CNN. In contrast, for graph filtration, connectivity matrices are constructed from 1D fMRI time series data using partial and marginal correlations. Persistent homology is computed for dimensions 0 and 1, and the top 10 most persistent features are used for classification via a stacked ensemble classifier. Furthermore, inter-subject Wasserstein distances between persistence diagrams as obtained through graph filtration for both homology dimensions are computed to assess statistically significant differences between the study groups. Though both methods gave decent performance in distinguishing for healthy and MCI, but, classification accuracy obtained using inter-ROI Wasserstein distance between persistent diagrams obtained using Vietoris-Rips filtration as feature significantly outperformed the graph filtration method that used the raw top ten most persistent homology features for classification. This is primarily because of its ability to work with flexible point clouds generated from rs-fMRI time series, rather than being constrained to fixed graph structures. This flexibility allows Vietoris-Rips to capture more detailed topological features across multiple dimensions- dimension-0 (connected components), 1 (loops), and 2 (voids), whereas graph filtration is limited to dimensions 0 and 1. Additionally, the use of inter-ROI Wasserstein distance in Vietoris-Rips filtration offers a more global comparison of brain connectivity patterns, enabling better characterization of the overall network topology. Neurodegenerative diseases like MCI affect large-scale brain networks, disrupting both local and global connectivity. Vietoris-Rips filtration captures these disruptions more effectively, making it a more robust tool for identifying subtle changes in brain network organization that are critical for early detection and classification of MCI subtypes. This enhanced sensitivity to complex network dynamics holds clinical relevance, as it may improve the accuracy of diagnostic models for MCI and other brain disorders.

**Figure 7:**
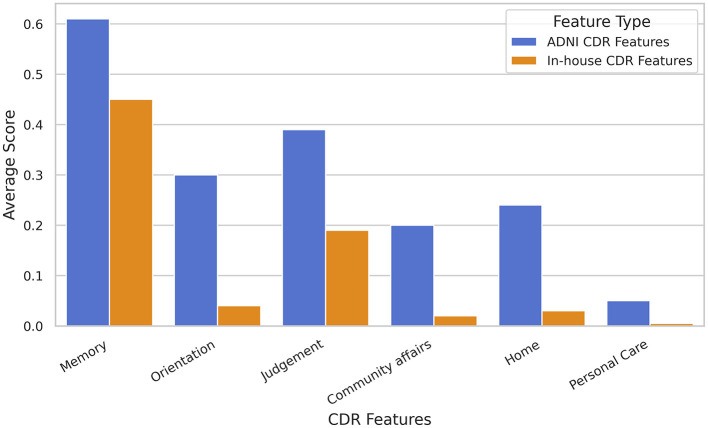
Illustrating the differences in six standard Clinical Dementia Rating (CDR) features between two distinct study populations: (i) the ADNI cohort and (ii) the in-house TLSA cohort.

Significant differences in classification accuracy are observed between the two distinct cohorts, which is one limitation of this study. These discrepancies arise from several factors. Populations from different regions or ethnicities may exhibit distinct demographic characteristics, genetic backgrounds, lifestyle factors, cultural practices, socioeconomic conditions, education levels, environmental exposures, and disease prevalence rates. Such differences can influence brain structure, function, and connectivity patterns, impacting the results of fMRI analyses. Figure 7 illustrates clear differences in six standard CDR features between the two cohorts. Additionally, variations in data acquisition protocols and imaging parameters may contribute to differences in functional connectivity patterns between datasets. To mitigate these limitations and strengthen the findings, further research should aim to validate results across more diverse and larger cohorts. Moreover, future scope may include employing advanced analytical approaches, such as multi-field topological analysis, may provide deeper insights into the influence of these variables and improve the generalizability of the results.

The present study underscores the clinical potential of persistent homology as a powerful tool for analyzing complex brain network dynamics. By leveraging persistent homology, we were able to capture intricate topological features that traditional methods might overlook, allowing for a more refined differentiation between distinct cognitive states. Our findings demonstrate the effectiveness of this approach in improving the classification accuracy of MCI subtypes, supporting its potential application in clinical diagnostics and decision-making. The novelty of our study lies in its application of persistent homology to identify subtle yet significant topological differences in brain connectivity, which could enhance the precision of MCI diagnoses, aid in tracking disease progression, and inform personalized treatment strategies. Moreover, the success of our model suggests that persistent homology could be extended to a wide range of clinical settings for improving diagnostic accuracy and patient outcomes across various neurological and medical conditions. Our study highlights the potential of integrating persistent homology into clinical workflows, offering the ability to enhance the precision and effectiveness of cognitive assessments. In future, this represents a significant advancement in the field of personalized medicine.

## 5 Conclusion

This study investigated the use of persistent homology techniques, specifically Vietoris-Rips and graph filtration methods, to examine complex brain network dynamics related to MCI. Persistent homology offers a powerful mathematical approach for analyzing topological features within data, and it shows potential in detecting subtle connectivity patterns that may indicate early cognitive decline. Through a comprehensive comparative analysis, our results suggest that Vietoris-Rips filtration may serve as an effective tool for diagnosing and monitoring MCI. This technique provides a refined view of brain connectivity alterations, with implications for advancing research and interventions aimed at early detection and management of cognitive decline.

## Data Availability

The raw data supporting the conclusions of this article will be made available by the authors, without undue reservation.

## Footnotes

^1^IRB approval statement: The inhouse TLSA data collection was approved by Institutional Ethics Committee at Centre for Brain Research vide number CBR-/42/IEC/2021-22.

^2^In algebraic topology, the topological features of a space are represented as *holes* or *cycles* in various dimensions. The number of *k*-dimensional holes in a *d*-dimensional simplicial complex (with *k* ≤ *d*) is denoted by the Betti number β_*k*_ or *H*_*k*_. Thus, 0-dimensional holes (β_0_*orH*_0_) correspond to connected components, 1-dimensional holes (β_1_*orH*_1_) represent tunnels (or loops) and 2-dimensional holes (β_2_*orH*_2_) are voids.
